# Successful treatment of a critically ill patient with acute respiratory distress syndrome from COVID-19 using mechanical ventilation strategy with low levels of positive end-expiratory pressure: A CARE-compliant case report

**DOI:** 10.1097/MD.0000000000023160

**Published:** 2020-12-04

**Authors:** Wei-guang Guo, Bin Fang, Yan-shan Xian, Zhi-hui Yu, Li-xin Zhou

**Affiliations:** Department of Critical Care Medicine of Foshan first people's Hospital, Foshan, 528000, Guangdong Province, China.

**Keywords:** COVID-19, low positive end-expiratory pressure level, low tidal volume, mechanical ventilation strategy, oxygenation status, pulmonary ventilation function

## Abstract

**Introduction:**

Acute respiratory distress syndrome **(**ARDS) secondary to COVID-19 is different from the ARDS caused by other infections. Conventional mechanical ventilation strategies using high levels of PEEP may not be beneficial and can even be harmful to patient with ARDS from COVID-19. So the ventilation strategies should be adjusted in order to improve the pulmonary ventilation function and oxygenation status, and outcomes of the patient.

**Patient concerns:**

Herein, we present a 76-year-old male patient with ARDS secondary to COVID-19. We describe our experience with mechanical ventilation strategy and the changes in respiratory mechanics in the patient during treatment.

**Diagnosis:**

The patient had tested positive for coronavirus (COVID-19) in nucleic acid test. Chest CT showed multiple ground glass shadows in both lungs.

**Interventions:**

The patient received mechanical ventilation with low tidal volume and low PEEP.

**Outcomes:**

After treatment, the patients condition, as well as oxygenation status was improved, and he tested negative for the coronavirus several times.

**Conclusion:**

This case demonstrated that the low tidal volume with low levels of PEEP ventilation strategy may be more suitable for ARDS from COVID-19.

## 1 Introduction

Since December 2019, coronavirus disease 2019 (COVID-19) broke out in Wuhan city, China, and is spreading around the world, WHO officials declared COVID-19 a global pandemic.^[1–4]^ The lungs are the main organs affected by COVID-19. COVID-19 is characterized by acute respiratory distress syndrome (ARDS) and progressive hypoxemia, which are important symptoms associated with poor prognosis.^[5,6]^ At present, the mechanical ventilation for patients with ARDS from COVID-19, mechanical ventilation is mainly performed according to the past experiences,^[7]^ i.e., using lung-protective ventilation strategy with high positive end-expiratory pressure (PEEP) levels and low tidal volume, but the efficacy is uncertain. More methods of respiratory support are needed to be explored in order to improve the prognosis of these patients. Informed consent was obtained from the patients for the publication of this study.

## 2 Case presentation

A 76-year-old male was admitted to the hospital on February 5, 2020 because of fever accompanied by dry cough for 5 days and shortness of breath for 2 days. The patient had a 10-year history of hypertension, coronary heart disease, hyperuricemia, and a 40-year history of smoking, and has not quit. He had recently traveled to Wuhan, China from January 14 to January 21. On February 1, 2020 (D1), he experienced fever, accompanied by fatigue, chills, and cough. Two days prior to admission (D3), he experienced shortness of breath, and the symptom was aggravated. Then the patient went to local hospital, and was tested positive for coronavirus (COVID-19) in nucleic acid tests. The patient was transferred to our hospital (D5) for further treatment.

**Figure 1:**
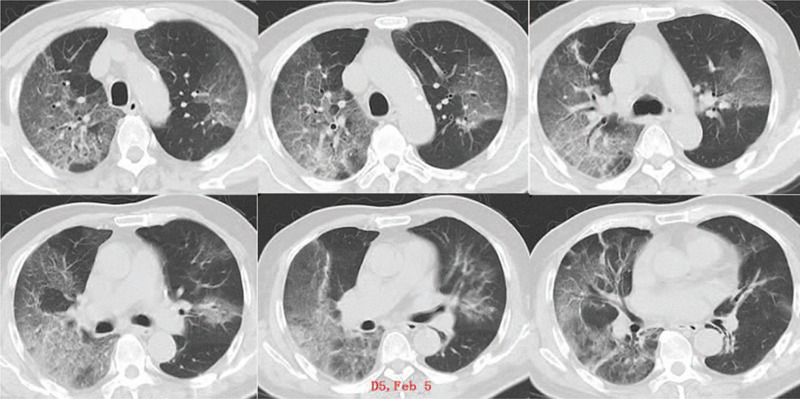
Chest CT images of the patient at admission on February 5, 2020 (D5).

**Figure 2:**
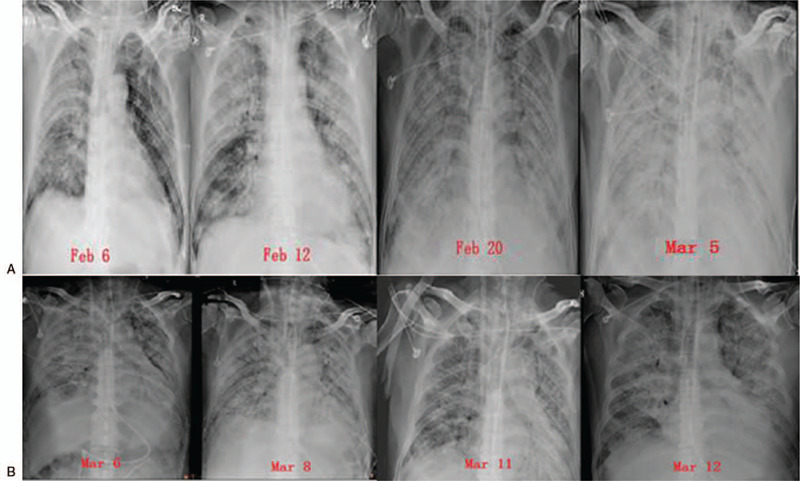
Chest X-ray images of the critically ill patient with acute respiratory distress syndrome from COVID-19 after receiving mechanical ventilation with a higher level of PEEP at the early stage of disease (A) and with a lower level of PEEP at the late stage of disease (B).

**Figure 3:**
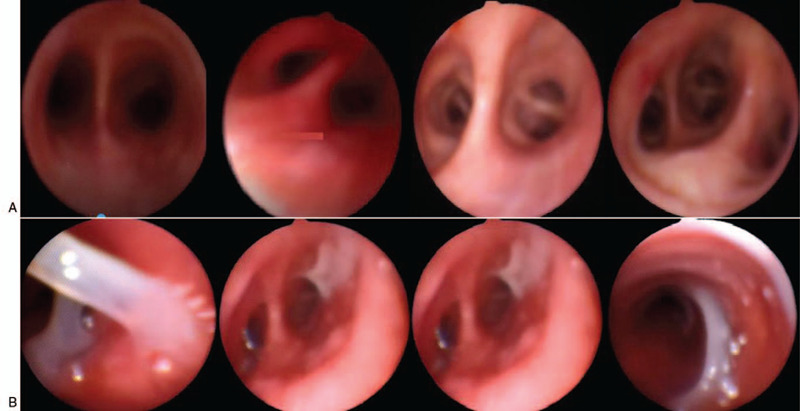
Fiberoptic bronchoscopy of the critically ill patient with acute respiratory distress syndrome from COVID-19 after receiving mechanical ventilation with a higher level of PEEP at the early stage of disease on February 13, 2020 (D13) (A) and with low levels of PEEP at the late stage of disease on March 6, 2020 (D35) (B).

**Figure 4:**
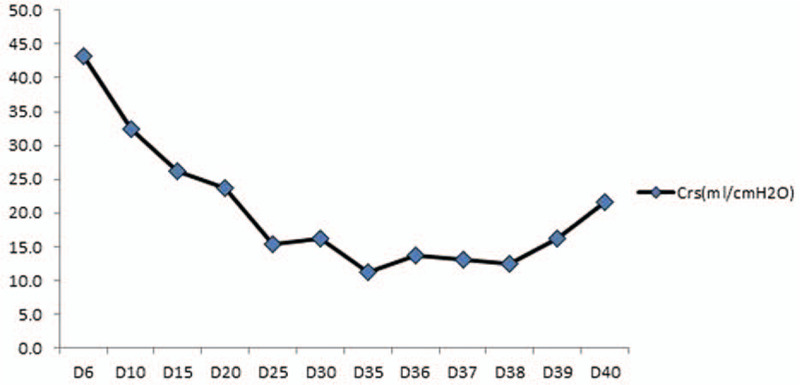
Changes in lung compliance in the critically ill patient with acute respiratory distress syndrome from COVID-19 receiving mechanical ventilation with low tidal volume (4 ml/kg).

Upon admission, blood routine test showed a white blood cell count of 4.96 × 10 ^9^ / L, lymphocyte count (L) of 0.36 × 10^9^/L (below the normal range), oxygenation index was 346 mm Hg. Chest CT showed multiple ground glass shadows in both lungs (Fig. 1). The patient was diagnosed with severe COVID-19. He received antiviral treatment (administration of abidol, lopinavir/ritonavir and IFN-γ), and HFNC oxygen therapy. But the patient still had obvious shortness of breath, and oxygenation status did not improve. Then the patient received noninvasive mechanical ventilation. On February 6 (D6), the patient received endotracheal intubation and mechanical ventilation with low tidal volume (Vt) and high PEEP (SIMV mode: Vt = 400 ml, PEEP = 10–15 cm H_2_O, frequency (mandatory respiratory rate per minute) = 15 breaths/minutes), and during mechanical ventilation, patient was kept prone position for 12–16 hours per day. On February 18 (D18), administration of antiviral drugs, lopinavir/ritonavir and abidol were stopped. During the treatment, pulmonary lesions of the patient had gradually worsened, the oxygenation was reduced, the chest X-ray showed increased pulmonary exudation (Fig. 2A), and pulmonary compliance was decreased. Fiberoptic bronchoscopy showed that there was no obvious sputum deposited in the main bronchi, and airway mucosa was smooth without edema and erosion (Fig. 3A). On February 18, the patient received venovenous extracorporeal membrane oxygenation (ECMO), but the patients lung condition was not improved, pulmonary compliance was further reduced. Then the mechanical ventilation strategy was adjusted (D35), optimal PEEP was set according to the low inflection point of the pressure-volume (P-V) curves, then measures in sputum expectoration was strengthened. After treatment, sputum volume was significantly increased, the sputum expectorated was white mucoid sputum bolts (Fig. 3B), and improvements in pulmonary ventilation function, pulmonary compliance, and pulmonary exudation were observed in the patient (Table 1, Fig. 2B, Fig. 4).

**Table 1:** Changes in respiratory mechanics in the critically ill patient with acute respiratory distress syndrome from COVID-19 during mechanical ventilation.

Days post onset of COVID-19	Tidal volume (Vt, ml/kg)	Peak pressure (Ppeak, cm H_2_O)	Plateau pressure (Pplat, cm H_2_O)	Positive end-expiratory pressure (PEEP, cm H_2_O)	_Δ_P (cm H_2_O)	Compliance of the respiratory system (Crs, ml/cm H_2_O)
D6	4	22	16	10	6	43.3
	6	26	20	10	10	39.0
D10	4	24	18	10	8	32.5
	6	30	22	10	12	32.5
D15	4	26	20	10	10	26.3
	6	36	28	10	18	21.6
D20	4	30	23	12	11	23.6
	6	33	25	12	13	20.0
D25	4	34	29	12	17	15.3
	6	39	32	12	20	12.2
D30	2	34	22	12	10	13.0
	4	37	28	12	16	16.2
D35	4	37	26	3	23	11.3
D36	4	30	22	3	19	13.7
D37	4	26	20	0	20	13.0
D38	4	26	21	0	21	12.4
D39	4	24	16	0	16	16.3
D40	4	20	12	0	12	21.7

Then the patients condition, as well as oxygenation status was improved and he tested negative for the coronavirus several times. On March 20, he was transferred out of the intensive care unit.

## 3 Discussion

In the present study, the disease course of the patient progressed rapidly to critical illness state, and he received endotracheal intubation. Treatment of patients with COVID-19 who developed ARDS was full of twists and turns. We found that the respiratory mechanics of the patient with ARDS from COVID-19 are different from patients with conventional ARDS. In the early stage, pulmonary compliance in the patient was normal, pulmonary ventilation function was also normal, or even higher, but the oxygenation status was poor, which reached the level that was considered as severe ARDS, and the respiratory mechanics and oxygenation status were not matched. We used early mechanical ventilation strategy involving a low tidal volume (4–6 ml/kg) and high levels of PEEP (10–15 cm H_2_O), combined with prone position ventilation. After treatment, initial oxygenation in the patient was improved, but the efficacy was difficult to maintain, and pulmonary compliance was decreased (Table 1), the patient ultimately received ECMO. The mechanical ventilation strategy was adjusted by using low tidal volume and low levels of PEEP. PEEP level was determined according to the low inflection point of the pressure-volume (P-V) curves, the lower inflection point defines the optimal PEEP level, and the PEEP levels (0–3 cm H_2_O) were lower. In addition, after we strengthened the measures in sputum expectoration, white viscous sputum was increased in the airways (Fig. 3B), pulmonary ventilation function, as well as pulmonary compliance of the patient were improved (Table 1).

In the present study, the patient had a long course of disease and due to the poor understanding of the pathophysiology of the disease at the early stage; we adopted lung-protective ventilation strategy, which led to worsening of the patients condition. The pathophysiologic characteristics of COVID-19 are inhomogeneous lung lesions and progressive lesions from outside to inside of the lungs.^[8]^ In the early stage, the degrees of pulmonary parenchymal, interstitial, and tracheal mucosal edema in the patient were not severe, the inflammatory response in the airways was not obvious, and very little sputum in the airways was observed by fiberoptic bronchoscopy. Regarding respiratory mechanics in the early phase of the disease, pulmonary compliance was normal, pulmonary ventilation function was also normal, or even higher. The mismatch between severe hypoxemia and better respiration mechanics is not common in patients with ARDS caused by other infections. This phenomenon is consistent with the observation of the characteristics of ARDS in patients with COVID-19 L-type by Gattinoni et al^[9]^ and has attracted the attention of researchers in multiple centers of China and researchers in Italy. This may be due to the loss of lung perfusion regulation and hypoxic pulmonary vasoconstriction. V/Q mismatch may also be due to that in the early phase, COVID-19 mainly damages alveolar epithelial cells, stimulates the aggregation of macrophages and cells with abundant cytoplasm, increases pulmonary interstitial inflammatory exudation,^[10]^ leading to interstitial pneumonia and lung diffusion capacity disorder. When the disease has progressed to a critical phase, in addition to severe hypoxemia, the patients showed decreased pulmonary compliance, worsening of V/Q mismatch, and lung volume reduction. Autopsy finding^[11]^ in a patient with COVID-19 showed that there were large amounts of off-white highly viscous fluids, and fiber cord in the lungs of the dead patient, and gelatinous mucus was found to be adhered to the trachea and bronchus cavity. Clinically, the inflammatory exudates in the lungs of patients were increased, and a large amounts of white gelatinous mucus were produced to block the alveoli and small and medium bronchi, causing pulmonary dysfunction and carbon dioxide retention, eventually, the pulmonary interstitial, alveolar lesions, and the lesions in small and medium airways were aggravated and lung consolidation occurred, lung compliance decreased significantly. Therefore, in the present study, ventilation strategy using low tidal volume and high levels of PEEP we adopted, can not improve the patients oxygenation status and prognosis, and on the contrary, high levels of PEEP prevent sputum expectoration, then the patients condition progressed to a worse condition. Therefore, mechanical ventilation with a high PEEP level is not recommended for treating patients with ARDS from COVID-19.

Lung recruitability has great guiding significance for the adjustment of mechanical ventilation strategies. In the present study, according to the observation from fiberoptic bronchoscopy, large amounts of white mucoid sputum bolts were produced during the disease course, the mucoid sputum was highly viscous and mainly concentrated in the alveoli and small airways, it is difficult to cough up by the patient himself. At this time, pulmonary compliance in the patient was decreased and lung recoilability was also markedly decreased. Therefore, using mechanical ventilation in the prone position and with high levels of PEEP cannot change the patients pulmonary ventilation function and lung diffusion capacity, and may easily lead to severe hemodynamic impairment and sputum retention. Therefore, it is very important to perform measures of sputum expectoration, such as adequate sputum clearance and bronchoalveolar lavage with fiberoptic bronchoscopy.

Mechanical ventilation with zero or low levels of PEEP violates the conventional ventilation strategy for ARDS; however, this strategy is suitable for ARDS from COVID-19. Lower PEEP ventilation can reduce airway pressure, and conform to the expiratory pressure gradient, which can contribute to sputum expectoration through thoracic and lung compliance, and patients own cough effort, reduce accumulation of mucus in the alveoli and small airways, and help to achieve recovery of pulmonary ventilation function and lung diffusion capacity, eventually help improve breathing and oxygenation status. Moreover, this strategy can significantly reduce ventilator-associated lung injury. In the present study, after the patient received low PEEP mechanical ventilation strategy during the late stage, sputum volume was significantly increased, the white mucoid sputum bolts could be gradually expectorated, and the pulmonary ventilation function and oxygenation status were both improved. Optimal PEEP titration can help us to understand the changes in actual respiratory mechanics in patients. Low-PEEP ventilation strategy may be a more appropriate for patients with ARDS from COVID. However, the pathophysiological process of lung injury in patients with COVID-19 is very complex, and there are individual differences. The mechanisms underlying the aggravation and progression of disease are still unclear, and there is a lack of pathological anatomical evidence from patients with COVID-19 at different phases. At present, there is no consensus on the application of mechanical ventilation in patients with COVID-19 who developed ARDS, which require further investigation.

The patients condition progressed and worsened rapidly, due to the existence of relative contraindications for ECMO therapy, such as advanced age, venous thrombosis of the lower extremities, airway hemorrhage and the timing of ECMO treatment was late. And due to the insufficient understanding of the pathophysiology of the disease at the early stage, the lung-protective ventilation strategy adopted led to prolonged time on mechanical ventilation, severe lung lesions, which made recovery difficult. In addition, due to the risk for infection, exposure to biological agents, and disease progression, CT scans of the patient with COVID-19 at different stages after admission was not obtained and compared, and lung and bronchial specimens were not collected and biopsy was not performed for verification.

In conclusion, the pathophysiological characteristics of ARDS from COVID-19 are different from that of the ARDS caused by other infections. The low tidal volume with low levels of PEEP ventilation strategy may be more suitable for patient with ARDS from COVID-19.

## Author contributions

**Conceptualization:** Li-xin Zhou.

**Data curation:** Wei-guang Guo, Bin Fang, Zhi-hui Yu.

**Formal analysis:** Bin Fang, Yan-shan Xian.

**Investigation:** Wei-guang Guo, Li-xin Zhou.

**Methodology:** Wei-guang Guo, Yan-shan Xian, Zhi-hui Yu, Li-xin Zhou.

**Resources:** Bin Fang, Yan-shan Xian, Zhi-hui Yu.

**Software:** Bin Fang.

**Supervision:** Zhi-hui Yu, Li-xin Zhou.

**Validation:** Yan-shan Xian.

**Writing – original draft:** Wei-guang Guo.

**Writing – review & editing:** Li-xin Zhou.
